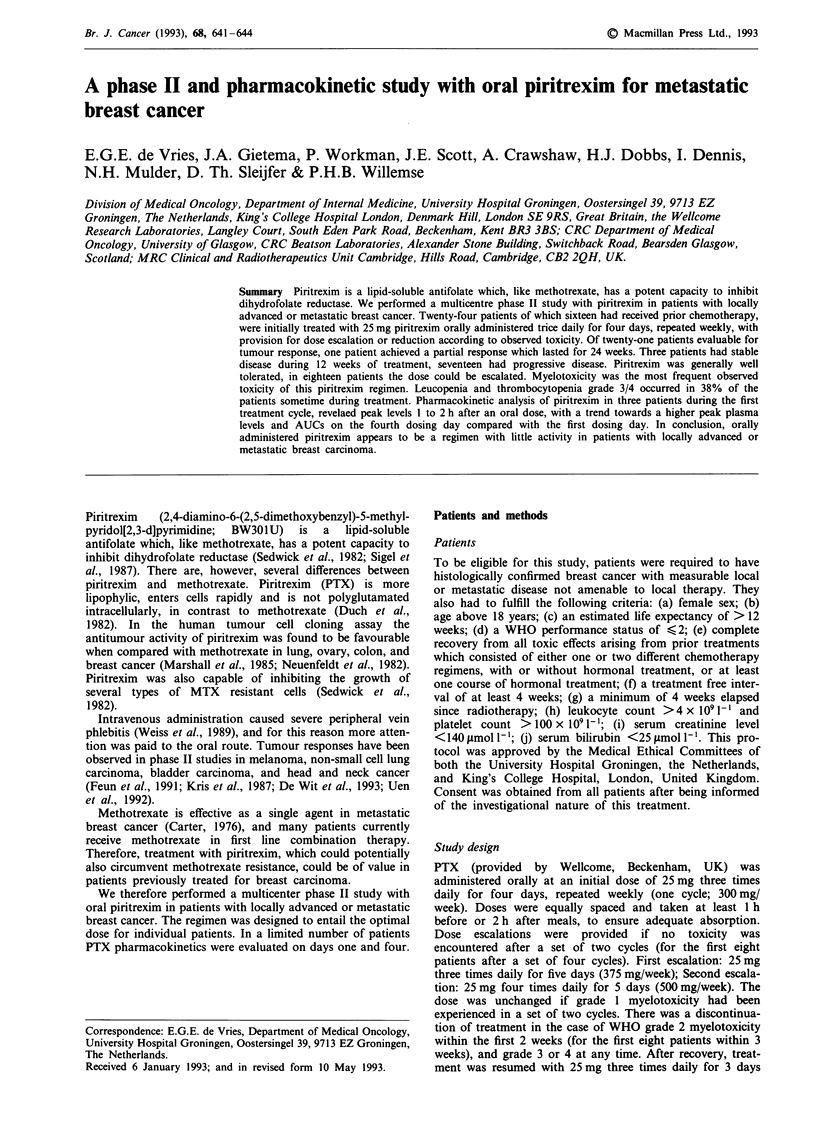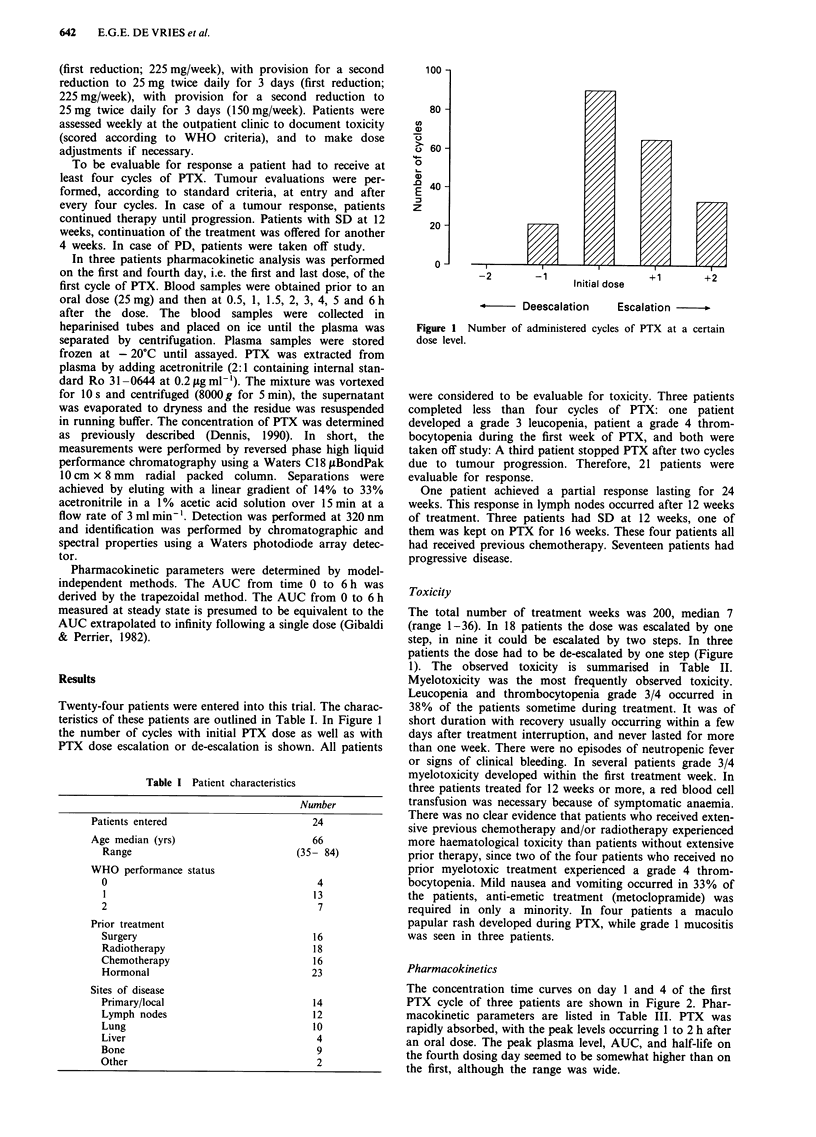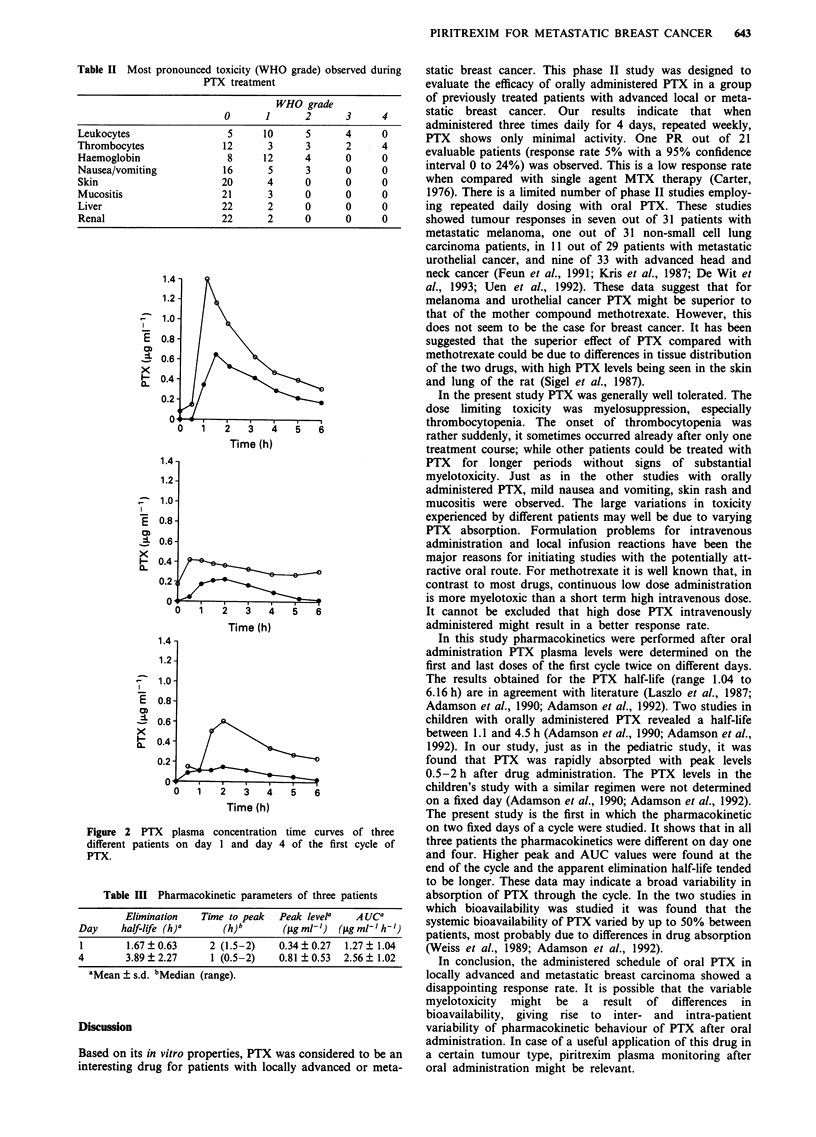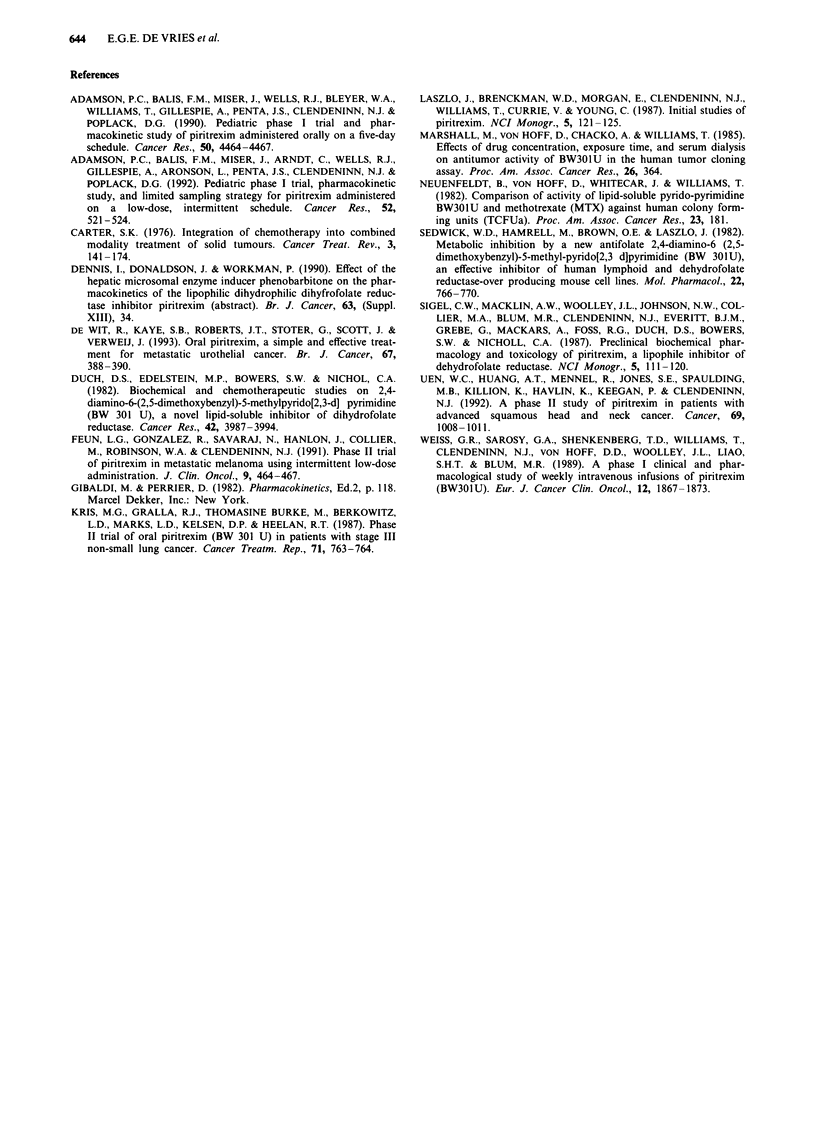# A phase II and pharmacokinetic study with oral piritrexim for metastatic breast cancer.

**DOI:** 10.1038/bjc.1993.400

**Published:** 1993-09

**Authors:** E. G. de Vries, J. A. Gietema, P. Workman, J. E. Scott, A. Crawshaw, H. J. Dobbs, I. Dennis, N. H. Mulder, D. T. Sleijfer, P. H. Willemse

**Affiliations:** Department of Internal Medicine, University Hospital Groningen, The Netherlands.

## Abstract

Piritrexim is a lipid-soluble antifolate which, like methotrexate, has a potent capacity to inhibit dihydrofolate reductase. We performed a multicentre phase II study with piritrexim in patients with locally advanced or metastatic breast cancer. Twenty-four patients of which sixteen had received prior chemotherapy, were initially treated with 25 mg piritrexim orally administered trice daily for four days, repeated weekly, with provision for dose escalation or reduction according to observed toxicity. Of twenty-one patients evaluable for tumour response, one patient achieved a partial response which lasted for 24 weeks. Three patients had stable disease during 12 weeks of treatment, seventeen had progressive disease. Pirtrexim was generally well tolerated, in eighteen patients the dose could be escalated. Myelotoxicity was the most frequent observed toxicity of this piritrexim regimen. Leucopenia and thrombocytopenia grade 3/4 occurred in 38% of the patients sometime during treatment. Pharmacokinetic analysis of piritrexim in three patients during the first treatment cycle, revealed peak levels 1 to 2 h after an oral dose, with a trend towards a higher peak plasma levels and AUCs on the fourth dosing day compared with the first dosing day. In conclusion, orally administered piritrexim appears to be a regimen with little activity in patients with locally advanced or metastatic breast carcinoma.


Br. J. Cancer (1993), 68, 641-644 © Macmillan Press Ltd., 1993

### A phase II and pharmacokinetic study with oral piritrexim for metastatic breast cancer

E.G.E. de Vries, J.A. Gietema, P. Workman, J.E. Scott, A. Crawshaw, H.J. Dobbs, I. Dennis, N.H. Mulder, D. Th. Sleijfer & P.H.B. Willemse

*Division of Medical Oncology, Department of Internal Medicine, University Hospital Groningen, Oostersingel 39, 9713 EZ Groningen, The Netherlands, King's College Hospital London, Denmark Hill, London SE 9RS, Great Britain, the Wellcome Research Laboratories, Langley Court, South Eden Park Road, Beckenham, Kent BR3 3BS; CRC Department of Medical Oncology, University of Glasgow, CRC Beatson Laboratories, Alexander Stone Building, Switchback Road, Bearsden Glasgow, Scotland; MRC Clinical and Radiotherapeutics Unit Cambridge, Hills Road, Cambridge, CB2 2QH, UK.*

**Summary** Piritrexim is a lipid-soluble antifolate which, like methotrexate, has a potent capacity to inhibit dihydrofolate reductase. We performed a multicentre phase II study with piritrexim in patients with locally advanced or metastatic breast cancer. Twenty-four patients of which sixteen had received prior chemotherapy, were initially treated with 25 mg piritrexim orally administered trice daily for four days, repeated weekly, with provision for dose escalation or reduction according to observed toxicity. Of twenty-one patients evaluable for tumour response, one patient achieved a partial response which lasted for 24 weeks. Three patients had stable disease during 12 weeks of treatment, seventeen had progressive disease. Piritrexim was generally well tolerated, in eighteen patients the dose could be escalated. Myelotoxicity was the most frequent observed toxicity of this piritrexim regimen. Leucopenia and thrombocytopenia grade 3/4 occurred in 38% of the patients sometime during treatment. Pharmacokinetic analysis of piritrexim in three patients during the first treatment cycle, revelaed peak levels 1 to 2 h after an oral dose, with a trend towards a higher peak plasma levels and AUCs on the fourth dosing day compared with the first dosing day. In conclusion, orally administered piritrexim appears to be a regimen with little activity in patients with locally advanced or metastatic breast carcinoma.

Piritrexim (2,4-diamino-6-(2,5-dimethoxybenzyl)-5-methyl-pyridol[2,3-d]pyrimidine; BW301U) is a lipid-soluble antifolate which, like methotrexate, has a potent capacity to inhibit dihydrofolate reductase (Sedwick *et al.*, 1982; Sigel *et al.*, 1987). There are, however, several differences between piritrexim and methotrexate. Piritrexim (PTX) is more lipophylic, enters cells rapidly and is not polyglutamated intracellularly, in contrast to methotrexate (Duch *et al.*, 1982). In the human tumour cell cloning assay the antitumour activity of piritrexim was found to be favourable when compared with methotrexate in lung, ovary, colon, and breast cancer (Marshall *et al.*, 1985; Neuenfeldt *et al.*, 1982). Piritrexim was also capable of inhibiting the growth of several types of MTX resistant cells (Sedwick *et al.*, 1982).

Intravenous administration caused severe peripheral vein phlebitis (Weiss *et al.*, 1989), and for this reason more attention was paid to the oral route. Tumour responses have been observed in phase II studies in melanoma, non-small cell lung carcinoma, bladder carcinoma, and head and neck cancer (Feun *et al.*, 1991; Kris *et al.*, 1987; De Wit *et al.*, 1993; Uen *et al.*, 1992).

Methotrexate is effective as a single agent in metastatic breast cancer (Carter, 1976), and many patients currently receive methotrexate in first line combination therapy. Therefore, treatment with piritrexim, which could potentially also circumvent methotrexate resistance, could be of value in patients previously treated for breast carcinoma.

We therefore performed a multicenter phase II study with oral piritrexim in patients with locally advanced or metastatic breast cancer. The regimen was designed to entail the optimal dose for individual patients. In a limited number of patients PTX pharmacokinetics were evaluated on days one and four.

Correspondence: E.G.E. de Vries, Department of Medical Oncology, University Hospital Groningen, Oostersingel 39, 9713 EZ Groningen, The Netherlands.
Received 6 January 1993; and in revised form 10 May 1993.

### Patients and methods

### Patients

To be eligible for this study, patients were required to have histologically confirmed breast cancer with measurable local or metastatic disease not amenable to local therapy. They also had to fulfill the following criteria: (a) female sex; (b) age above 18 years; (c) an estimated life expectancy of $>12$ weeks; (d) a WHO performance status of $\leqslant 2$; (e) complete recovery from all toxic effects arising from prior treatments which consisted of either one or two different chemotherapy regimens, with or without hormonal treatment, or at least one course of hormonal treatment; (f) a treatment free interval of at least 4 weeks; (g) a minimum of 4 weeks elapsed since radiotherapy; (h) leukocyte count $>4 \times 10^9\,l^{-1}$ and platelet count $>100 \times 10^9\,l^{-1}$; (i) serum creatinine level $<140\,\mu mol\,l^{-1}$; (j) serum bilirubin $<25\,\mu mol\,l^{-1}$. This protocol was approved by the Medical Ethical Committees of both the University Hospital Groningen, the Netherlands, and King's College Hospital, London, United Kingdom. Consent was obtained from all patients after being informed of the investigational nature of this treatment.

### Study design

PTX (provided by Wellcome, Beckenham, UK) was administered orally at an initial dose of 25 mg three times daily for four days, repeated weekly (one cycle; 300 mg/week). Doses were equally spaced and taken at least 1 h before or 2 h after meals, to ensure adequate absorption. Dose escalations were provided if no toxicity was encountered after a set of two cycles (for the first eight patients after a set of four cycles). First escalation: 25 mg three times daily for five days (375 mg/week); Second escalation: 25 mg four times daily for 5 days (500 mg/week). The dose was unchanged if grade 1 myelotoxicity had been experienced in a set of two cycles. There was a discontinuation of treatment in the case of WHO grade 2 myelotoxicity within the first 2 weeks (for the first eight patients within 3 weeks), and grade 3 or 4 at any time. After recovery, treatment was resumed with 25 mg three times daily for 3 days

(first reduction; 225 mg/week), with provision for a second reduction to 25 mg twice daily for 3 days (first reduction; 225 mg/week), with provision for a second reduction to 25 mg twice daily for 3 days (150 mg/week). Patients were assessed weekly at the outpatient clinic to document toxicity (scored according to WHO criteria), and to make dose adjustments if necessary.

To be evaluable for response a patient had to receive at least four cycles of PTX. Tumour evaluations were performed, according to standard criteria, at entry and after every four cycles. In case of a tumour response, patients continued therapy until progression. Patients with SD at 12 weeks, continuation of the treatment was offered for another 4 weeks. In case of PD, patients were taken off study.

In three patients pharmacokinetic analysis was performed on the first and fourth day, i.e. the first and last dose, of the first cycle of PTX. Blood samples were obtained prior to an oral dose (25 mg) and then at 0.5, 1, 1.5, 2, 3, 4, 5 and 6 h after the dose. The blood samples were collected in heparinised tubes and placed on ice until the plasma was separated by centrifugation. Plasma samples were stored frozen at − 20°C until assayed. PTX was extracted from plasma by adding acetronitrile (2:1 containing internal standard Ro 31–0644 at 0.2 $\mu g\ ml^{-1}$). The mixture was vortexed for 10 s and centrifuged (8000 *g* for 5 min), the supernatant was evaporated to dryness and the residue was resuspended in running buffer. The concentration of PTX was determined as previously described (Dennis, 1990). In short, the measurements were performed by reversed phase high liquid performance chromatography using a Waters C18 μBondPak 10 cm × 8 mm radial packed column. Separations were achieved by eluting with a linear gradient of 14% to 33% acetonitrile in a 1% acetic acid solution over 15 min at a flow rate of 3 $ml\ min^{-1}$. Detection was performed at 320 nm and identification was performed by chromatographic and spectral properties using a Waters photodiode array detector.

Pharmacokinetic parameters were determined by model-independent methods. The AUC from time 0 to 6 h was derived by the trapezoidal method. The AUC from 0 to 6 h measured at steady state is presumed to be equivalent to the AUC extrapolated to infinity following a single dose (Gibaldi & Perrier, 1982).

### Results

Twenty-four patients were entered into this trial. The characteristics of these patients are outlined in Table I. In Figure 1 the number of cycles with initial PTX dose as well as with PTX dose escalation or de-escalation is shown. All patients

**Table I** Patient characteristics

| | *Number* |
|---|---|
| Patients entered | 24 |
| Age median (yrs) | 66 |
| Range | (35- 84) |
| WHO performance status | |
| 0 | 4 |
| 1 | 13 |
| 2 | 7 |
| Prior treatment | |
| Surgery | 16 |
| Radiotherapy | 18 |
| Chemotherapy | 16 |
| Hormonal | 23 |
| Sites of disease | |
| Primary/local | 14 |
| Lymph nodes | 12 |
| Lung | 10 |
| Liver | 4 |
| Bone | 9 |
| Other | 2 |

**Figure 1** Number of administered cycles of PTX at a certain dose level.

were considered to be evaluable for toxicity. Three patients completed less than four cycles of PTX: one patient developed a grade 3 leucopenia, patient a grade 4 thrombocytopenia during the first week of PTX, and both were taken off study: A third patient stopped PTX after two cycles due to tumour progression. Therefore, 21 patients were evaluable for response.

One patient achieved a partial response lasting for 24 weeks. This response in lymph nodes occurred after 12 weeks of treatment. Three patients had SD at 12 weeks, one of them was kept on PTX for 16 weeks. These four patients all had received previous chemotherapy. Seventeen patients had progressive disease.

### *Toxicity*

The total number of treatment weeks was 200, median 7 (range 1–36). In 18 patients the dose was escalated by one step, in nine it could be escalated by two steps. In three patients the dose had to be de-escalated by one step (Figure 1). The observed toxicity is summarised in Table II. Myelotoxicity was the most frequently observed toxicity. Leucopenia and thrombocytopenia grade 3/4 occurred in 38% of the patients sometime during treatment. It was of short duration with recovery usually occurring within a few days after treatment interruption, and never lasted for more than one week. There were no episodes of neutropenic fever or signs of clinical bleeding. In several patients grade 3/4 myelotoxicity developed within the first treatment week. In three patients treated for 12 weeks or more, a red blood cell transfusion was necessary because of symptomatic anaemia. There was no clear evidence that patients who received extensive previous chemotherapy and/or radiotherapy experienced more haematological toxicity than patients without extensive prior therapy, since two of the four patients who received no prior myelotoxic treatment experienced a grade 4 thrombocytopenia. Mild nausea and vomiting occurred in 33% of the patients, anti-emetic treatment (metoclopramide) was required in only a minority. In four patients a maculo papular rash developed during PTX, while grade 1 mucositis was seen in three patients.

### *Pharmacokinetics*

The concentration time curves on day 1 and 4 of the first PTX cycle of three patients are shown in Figure 2. Pharmacokinetic parameters are listed in Table III. PTX was rapidly absorbed, with the peak levels occurring 1 to 2 h after an oral dose. The peak plasma level, AUC, and half-life on the fourth dosing day seemed to be somewhat higher than on the first, although the range was wide.

**Table II** Most pronounced toxicity (WHO grade) observed during PTX treatment

| | WHO grade 0 | 1 | 2 | 3 | 4 |
|---|---|---|---|---|---|
| Leukocytes | 5 | 10 | 5 | 4 | 0 |
| Thrombocytes | 12 | 3 | 3 | 2 | 4 |
| Haemoglobin | 8 | 12 | 4 | 0 | 0 |
| Nausea/vomiting | 16 | 5 | 3 | 0 | 0 |
| Skin | 20 | 4 | 0 | 0 | 0 |
| Mucositis | 21 | 3 | 0 | 0 | 0 |
| Liver | 22 | 2 | 0 | 0 | 0 |
| Renal | 22 | 2 | 0 | 0 | 0 |

**Figure 2** PTX plasma concentration time curves of three different patients on day 1 and day 4 of the first cycle of PTX.

**Table III** Pharmacokinetic parameters of three patients

| Day | Elimination half-life (h)[a] | Time to peak (h)[b] | Peak level[a] ($\mu g\ ml^{-1}$) | AUC[a] ($\mu g\ ml^{-1}\ h^{-1}$) |
|---|---|---|---|---|
| 1 | 1.67 ± 0.63 | 2 (1.5–2) | 0.34 ± 0.27 | 1.27 ± 1.04 |
| 4 | 3.89 ± 2.27 | 1 (0.5–2) | 0.81 ± 0.53 | 2.56 ± 1.02 |

[a]Mean ± s.d. [b]Median (range).

### Discussion

Based on its *in vitro* properties, PTX was considered to be an interesting drug for patients with locally advanced or metastatic breast cancer. This phase II study was designed to evaluate the efficacy of orally administered PTX in a group of previously treated patients with advanced local or metastatic breast cancer. Our results indicate that when administered three times daily for 4 days, repeated weekly, PTX shows only minimal activity. One PR out of 21 evaluable patients (response rate 5% with a 95% confidence interval 0 to 24%) was observed. This is a low response rate when compared with single agent MTX therapy (Carter, 1976). There is a limited number of phase II studies employing repeated daily dosing with oral PTX. These studies showed tumour responses in seven out of 31 patients with metastatic melanoma, one out of 31 non-small cell lung carcinoma patients, in 11 out of 29 patients with metastatic urothelial cancer, and nine of 33 with advanced head and neck cancer (Feun *et al.*, 1991; Kris *et al.*, 1987; De Wit *et al.*, 1993; Uen *et al.*, 1992). These data suggest that for melanoma and urothelial cancer PTX might be superior to that of the mother compound methotrexate. However, this does not seem to be the case for breast cancer. It has been suggested that the superior effect of PTX compared with methotrexate could be due to differences in tissue distribution of the two drugs, with high PTX levels being seen in the skin and lung of the rat (Sigel *et al.*, 1987).

In the present study PTX was generally well tolerated. The dose limiting toxicity was myelosuppression, especially thrombocytopenia. The onset of thrombocytopenia was rather suddenly, it sometimes occurred already after only one treatment course; while other patients could be treated with PTX for longer periods without signs of substantial myelotoxicity. Just as in the other studies with orally administered PTX, mild nausea and vomiting, skin rash and mucositis were observed. The large variations in toxicity experienced by different patients may well be due to varying PTX absorption. Formulation problems for intravenous administration and local infusion reactions have been the major reasons for initiating studies with the potentially attractive oral route. For methotrexate it is well known that, in contrast to most drugs, continuous low dose administration is more myelotoxic than a short term high intravenous dose. It cannot be excluded that high dose PTX intravenously administered might result in a better response rate.

In this study pharmacokinetics were performed after oral administration PTX plasma levels were determined on the first and last doses of the first cycle twice on different days. The results obtained for the PTX half-life (range 1.04 to 6.16 h) are in agreement with literature (Laszlo *et al.*, 1987; Adamson *et al.*, 1990; Adamson *et al.*, 1992). Two studies in children with orally administered PTX revealed a half-life between 1.1 and 4.5 h (Adamson *et al.*, 1990; Adamson *et al.*, 1992). In our study, just as in the pediatric study, it was found that PTX was rapidly absorpted with peak levels 0.5–2 h after drug administration. The PTX levels in the children's study with a similar regimen were not determined on a fixed day (Adamson *et al.*, 1990; Adamson *et al.*, 1992). The present study is the first in which the pharmacokinetic on two fixed days of a cycle were studied. It shows that in all three patients the pharmacokinetics were different on day one and four. Higher peak and AUC values were found at the end of the cycle and the apparent elimination half-life tended to be longer. These data may indicate a broad variability in absorption of PTX through the cycle. In the two studies in which bioavailability was studied it was found that the systemic bioavailability of PTX varied by up to 50% between patients, most probably due to differences in drug absorption (Weiss *et al.*, 1989; Adamson *et al.*, 1992).

In conclusion, the administered schedule of oral PTX in locally advanced and metastatic breast carcinoma showed a disappointing response rate. It is possible that the variable myelotoxicity might be a result of differences in bioavailability, giving rise to inter- and intra-patient variability of pharmacokinetic behaviour of PTX after oral administration. In case of a useful application of this drug in a certain tumour type, piritrexim plasma monitoring after oral administration might be relevant.

### References

ADAMSON, P.C., BALIS, F.M., MISER, J., WELLS, R.J., BLEYER, W.A., WILLIAMS, T., GILLESPIE, A., PENTA, J.S., CLENDENINN, N.J. & POPLACK, D.G. (1990). Pediatric phase I trial and pharmacokinetic study of piritrexim administered orally on a five-day schedule. *Cancer Res.*, **50**, 4464–4467.

ADAMSON, P.C., BALIS, F.M., MISER, J., ARNDT, C., WELLS, R.J., GILLESPIE, A., ARONSON, L., PENTA, J.S., CLENDENINN, N.J. & POPLACK, D.G. (1992). Pediatric phase I trial, pharmacokinetic study, and limited sampling strategy for piritrexim administered on a low-dose, intermittent schedule. *Cancer Res.*, **52**, 521–524.

CARTER, S.K. (1976). Integration of chemotherapy into combined modality treatment of solid tumours. *Cancer Treat. Rev.*, **3**, 141–174.

DENNIS, I., DONALDSON, J. & WORKMAN, P. (1990). Effect of the hepatic microsomal enzyme inducer phenobarbitone on the pharmacokinetics of the lipophilic dihydrophilic dihyfrofolate reductase inhibitor piritrexim (abstract). *Br. J. Cancer*, **63**, (Suppl. XIII), 34.

DE WIT, R., KAYE, S.B., ROBERTS, J.T., STOTER, G., SCOTT, J. & VERWEIJ, J. (1993). Oral piritrexim, a simple and effective treatment for metastatic urothelial cancer. *Br. J. Cancer*, **67**, 388–390.

DUCH, D.S., EDELSTEIN, M.P., BOWERS, S.W. & NICHOL, C.A. (1982). Biochemical and chemotherapeutic studies on 2,4-diamino-6-(2,5-dimethoxybenzyl)-5-methylpyrido[2,3-d] pyrimidine (BW 301 U), a novel lipid-soluble inhibitor of dihydrofolate reductase. *Cancer Res.*, **42**, 3987–3994.

FEUN, L.G., GONZALEZ, R., SAVARAJ, N., HANLON, J., COLLIER, M., ROBINSON, W.A. & CLENDENINN, N.J. (1991). Phase II trial of piritrexim in metastatic melanoma using intermittent low-dose administration. *J. Clin. Oncol.*, **9**, 464–467.

GIBALDI, M. & PERRIER, D. (1982). *Pharmacokinetics*, Ed.2, p. 118. Marcel Dekker, Inc.: New York.

KRIS, M.G., GRALLA, R.J., THOMASINE BURKE, M., BERKOWITZ, L.D., MARKS, L.D., KELSEN, D.P. & HEELAN, R.T. (1987). Phase II trial of oral piritrexim (BW 301 U) in patients with stage III non-small lung cancer. *Cancer Treatm. Rep.*, **71**, 763–764.

LASZLO, J., BRENCKMAN, W.D., MORGAN, E., CLENDENINN, N.J., WILLIAMS, T., CURRIE, V. & YOUNG, C. (1987). Initial studies of piritrexim. *NCI Monogr.*, **5**, 121–125.

MARSHALL, M., VON HOFF, D., CHACKO, A. & WILLIAMS, T. (1985). Effects of drug concentration, exposure time, and serum dialysis on antitumor activity of BW301U in the human tumor cloning assay. *Proc. Am. Assoc. Cancer Res.*, **26**, 364.

NEUENFELDT, B., VON HOFF, D., WHITECAR, J. & WILLIAMS, T. (1982). Comparison of activity of lipid-soluble pyrido-pyrimidine BW301U and methotrexate (MTX) against human colony forming units (TCFUa). *Proc. Am. Assoc. Cancer Res.*, **23**, 181.

SEDWICK, W.D., HAMRELL, M., BROWN, O.E. & LASZLO, J. (1982). Metabolic inhibition by a new antifolate 2,4-diamino-6 (2,5-dimethoxybenzyl)-5-methyl-pyrido[2,3 d]pyrimidine (BW 301U), an effective inhibitor of human lymphoid and dehydrofolate reductase-over producing mouse cell lines. *Mol. Pharmacol.*, **22**, 766–770.

SIGEL, C.W., MACKLIN, A.W., WOOLLEY, J.L., JOHNSON, N.W., COLLIER, M.A., BLUM, M.R., CLENDENINN, N.J., EVERITT, B.J.M., GREBE, G., MACKARS, A., FOSS, R.G., DUCH, D.S., BOWERS, S.W. & NICHOLL, C.A. (1987). Preclinical biochemical pharmacology and toxicology of piritrexim, a lipophile inhibitor of dehydrofolate reductase. *NCI Monogr.*, **5**, 111–120.

UEN, W.C., HUANG, A.T., MENNEL, R., JONES, S.E., SPAULDING, M.B., KILLION, K., HAVLIN, K., KEEGAN, P. & CLENDENINN, N.J. (1992). A phase II study of piritrexim in patients with advanced squamous head and neck cancer. *Cancer*, **69**, 1008–1011.

WEISS, G.R., SAROSY, G.A., SHENKENBERG, T.D., WILLIAMS, T., CLENDENINN, N.J., VON HOFF, D.D., WOOLLEY, J.L., LIAO, S.H.T. & BLUM, M.R. (1989). A phase I clinical and pharmacological study of weekly intravenous infusions of piritrexim (BW301U). *Eur. J. Cancer Clin. Oncol.*, **12**, 1867–1873.